# Pharmacomicrobiomics in Psoriasis: Microbiome–Drug Interactions Across Systemic Treatments

**DOI:** 10.3390/life16030415

**Published:** 2026-03-04

**Authors:** Umberto Santaniello, Luca Mastorino, Valentina Pala, Francois Rosset, Orsola Crespi, Pietro Quaglino, Simone Ribero

**Affiliations:** 1 Section of Dermatology, Department of Medical Sciences, University of Turin, Via Cherasco 23, 10126 Turin, Italy; 2 Department of Dermatology, Beauregard Hospital, Azienda USL della Valle d’Aosta, 11100 Aosta, Italy

**Keywords:** psoriasis, pharmacomicrobiomics, microbiome, gut microbiota, skin microbiota, drug metabolism, biologic therapy, methotrexate, probiotics, fecal microbiota transplantation (FMT)

## Abstract

Psoriasis is a chronic immune-mediated skin disease with highly variable responses to systemic therapies. Emerging evidence highlights the microbiome as a potential modulator of drug efficacy and toxicity. Gut bacteria can enzymatically metabolize drugs, such as methotrexate, altering bioavailability and therapeutic outcomes, while microbial metabolites—including short-chain fatty acids, branched-chain amino acids, and tryptophan derivatives—shape host immunity and barrier integrity, influencing drug action. Baseline microbial signatures have been linked to treatment response, potentially predicting anti-TNF or IL-17 inhibitor efficacy. Systemic therapies themselves reshape microbial communities: IL-17 blockade induces broad shifts in gut and skin microbiota, whereas cyclosporine and anti-TNF agents exert subtler effects. Small molecules such as apremilast and fumarates may reduce fungal overgrowth and influence microbial composition, whereas data on JAK/TYK2 inhibitors remain limited. Notably, current evidence exhibits a literature bias toward the gut microbiota, while the roles of the oral and skin axes remain understudied. Adjunctive microbiome-directed interventions, including probiotics and fecal microbiota transplantation, have demonstrated potential to enhance treatment outcomes by promoting anti-inflammatory taxa and restoring barrier function. Despite these promising findings, current evidence is heterogeneous, often limited by small sample sizes, short follow-up, and variable methodology. Integrating pharmacomicrobiomics data with clinical, genetic, and multi-omics profiling could enable precision medicine approaches in psoriasis, allowing therapy selection tailored to individual microbial and metabolic signatures. Future research should focus on longitudinal, multicenter studies to identify actionable microbial biomarkers, clarify mechanistic interactions between drugs, microbes, and host immunity, and evaluate microbiome-targeted adjuncts in randomized trials. Understanding the bidirectional crosstalk between systemic therapies and the microbiome may transform psoriasis management, improving efficacy, reducing adverse events, and enabling durable, personalized responses.

## 1. Introduction

Psoriasis is a chronic, inflammatory, immune-mediated skin disease driven by dysregulated cytokine pathways (IL-6, INF, TNF, IL-17, IL-23), and influenced by genetic and environmental factors [1]. Patient responses to therapies vary greatly, traditionally attributed to host genetics and environment. Recently, the microbiome has been proposed as a key factor influencing drug response [2]. Host gut and skin microbes can enzymatically modify drugs, altering their absorption and metabolism, while systemic immunomodulators in turn reshape microbial communities [3,4]. Pharmacomicrobiomics examines how microbiota affect drug pharmacokinetics (PK) and or pharmacodynamics (PD) and how therapies alter microbial communities [5]. Such interactions may be crucial in psoriasis: for example, gut microbiota composition and function not only differ in psoriasis patients (e.g., reduced short-chain fatty acid producers and lowered *Akkermansia muciniphila* abundance), but also change dynamically with therapy, potentially affecting treatment response [6,7]. In fact, microbial enzymes can directly metabolize drugs (e.g., bacterial carboxypeptidase degradation of methotrexate), microbial metabolites can modulate drug targets (e.g., short-chain fatty acids influencing IL-17/Treg balance), and therapies can reshape the microbiome in ways that affect both efficacy and safety (e.g., IL-17 inhibitor-induced gut dysbiosis correlating with inflammatory bowel disease risk) [8,9]. The aim of this review is to critically synthesize current evidence on pharmacomicrobiomics in psoriasis, with a specific focus on how gut, skin, and oral microbiota influence the pharmacokinetics, pharmacodynamics, efficacy, and safety of systemic therapies. By integrating mechanistic insights with clinical data, we aim to clarify which microbiome–drug interactions are biologically plausible, clinically relevant, and potentially actionable, while highlighting current gaps that preclude routine clinical implementation.

## 2. Methods

We performed a comprehensive narrative literature review of English-language publications (PubMed, PMC, Web of Science) up to July 2025. The search was conducted using targeted combinations of keywords and Boolean operators to ensure a broad yet specific coverage of the field. To ensure the inclusion of the most recent and impactful evidence, additional key studies published through early 2026 were manually identified and integrated during the revision process. The search strategy integrated terms related to psoriasis (e.g., “psoriasis”, “psoriatic”) with a broad range of microbiome-related descriptors, including “microbiome”, “microbiota”, “gut bacteria”, “dysbiosis”, “gut flora”, “skin microbiome”, and “oral microbiota”. To capture the pharmacomicrobiomic evidence, these were cross-referenced with specific therapeutic agents and classes, such as “methotrexate”, “cyclosporine”, “acitretin”, “apremilast”, “deucravacitinib”, “JAK inhibitor”, and “biologic*”. Detailed searches were performed for individual molecules, including TNF-α inhibitors (adalimumab, infliximab, etanercept, certolizumab pegol), IL-17 inhibitors (secukinumab, ixekizumab, bimekizumab, brodalumab), IL-23 inhibitors (guselkumab, risankizumab, tildrakizumab), and the IL-12/23 inhibitor (ustekinumab). Furthermore, terms such as “pharmacokinetics”, “pharmacodynamics”, “drug metabolism”, “treatment response”, “biomarker”, “probiotic*”, and “fecal microbiota transplant” (FMT) were employed to identify mechanistic and clinical outcomes. We included original research (clinical trials, cohort, and cross-sectional studies), preclinical models providing mechanistic insights, and systematic reviews or meta-analyses. Exclusion criteria focused on (1) articles not available in full text, (2) case reports with insufficient mechanistic data, (3) studies focusing exclusively on topical therapies without systemic relevance, and (4) duplicate publications. Outcomes of interest were the effects of baseline microbiota on drug PK/PD and response; the effects of drugs on microbial composition/function; identified mechanisms; microbial biomarkers of response/toxicity; and therapeutic implications. Evidence was synthesized narratively and organized thematically by therapeutic class.

## 3. Gut, Oral and Skin Microbiota in Psoriasis

### 3.1. Gut Dysbiosis in Psoriasis

**Figure 1:**
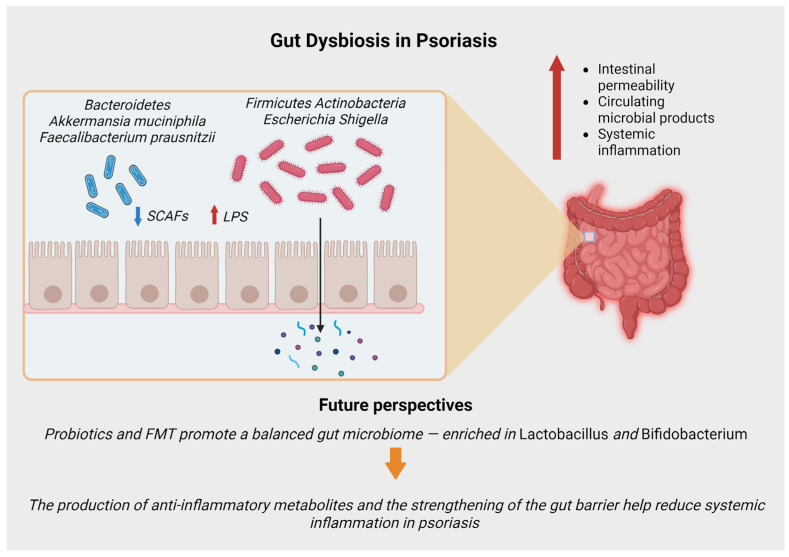
The psoriatic gut microbiome is characterized by a significant loss of alpha-diversity and functional dysbiosis. Key beneficial taxa, specifically mucin-degrading *Akkermansia muciniphila* and butyrate-producing *Faecalibacterium prausnitzii*, are depleted, while pro-inflammatory pathobionts (e.g., *Escherichia–Shigella*) are expanded. This imbalance compromises gut barrier integrity (“leaky gut”), facilitating the translocation of Microbe-Associated Molecular Patterns (MAMPs), such as lipopolysaccharide (LPS), into the systemic circulation. Concurrently, the reduction in short-chain fatty acids (SCFAs) impairs the induction of regulatory T cells (Tregs), shifting the immune balance toward a Th1/Th17-dominant inflammatory profile. Emerging interventions—such as probiotics and Fecal micriobiot transplant (FMT)—aim to restore this microbial equilibrium and enhance therapeutic efficacy.

The psoriatic gut microbiome exhibits consistent dysbiosis (Figure 1, Table 1). Compared to healthy individuals, psoriasis patients show altered microbial taxa: for example, decreased abundance of *Bacteroidetes* (including *Bacteroides* spp.) and increased *Firmicutes* and *Actinobacteria* have been reported [10]. In particular, an expansion of pathobionts such as *Escherichia-Shigella* has been observed in untreated psoriasis and correlates with higher pro-inflammatory markers [10]. Conversely, a reduction in mucin-degrading bacteria like *Akkermansia muciniphila* and butyrate-producers such as *Faecalibacterium prausnitzii* has been consistently reported [6,7]. This dysbiosis may increase intestinal permeability and systemic inflammation; in fact, gut barrier defects and elevated circulating microbial products (e.g., lipopolysaccharide) have been detected in psoriasis, supporting a disrupted gut barrier [11]. This barrier dysfunction is further exacerbated by functional metabolic shifts. The psoriatic microbiome is characterized by a marked reduction in the production of short-chain fatty acids (SCFAs), particularly acetate and butyrate. Under physiological conditions, SCFAs are crucial for maintaining barrier integrity and inducing regulatory T cells (Tregs) to suppress inflammation. Their depletion, combined with the enrichment of pro-inflammatory bacterial components like lipopolysaccharide (LPS) from Gram-negative organisms, drives the immune system toward a Th1/Th17-dominant profile, thereby amplifying the psoriatic inflammatory cascade [6].

### 3.2. Cutaneous Microbiota in Psoriasis

**Figure 2:**
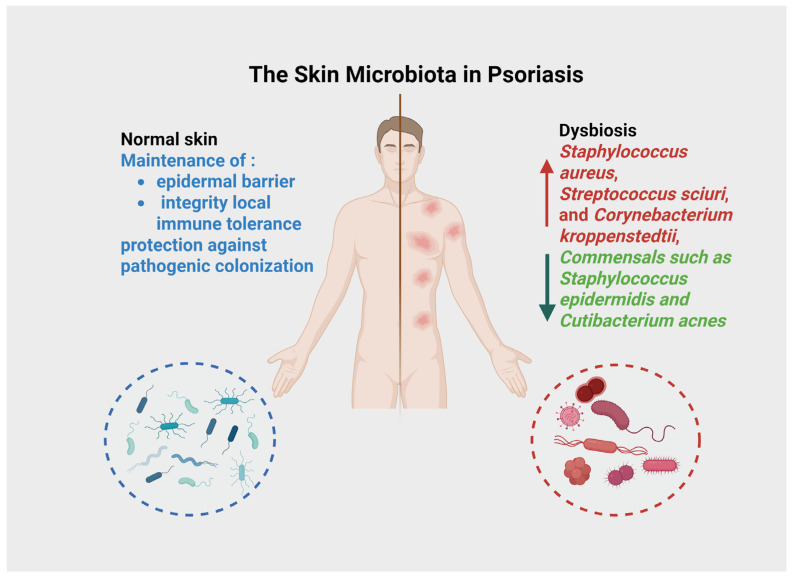
The skin microbiota is essential for epidermal barrier integrity, immune tolerance, and defense against pathogens. In psoriasis, this microbial community shows reduced diversity and a shift toward increased *Staphylococcus aureus*, *Streptococcus sciuri*, and *Corynebacterium kroppenstedtii*, with a loss of commensals like *Staphylococcus epidermidis* and *Cutibacterium acnes*. These alterations are more evident in lesional than in non-lesional skin, indicating disease-associated dysbiosis.

The skin microbiota plays a central role in maintaining epidermal barrier integrity, local immune tolerance, and protection against pathogenic colonization (Figure 2). It comprises a dynamic community of bacteria, fungi, viruses, and mites, with dominant genera including *Staphylococcus*, *Cutibacterium*, and *Corynebacterium* [12]. Unlike the gut microbiota, which has been extensively studied in psoriasis, the cutaneous microbial ecosystem has only recently garnered attention in relation to psoriatic inflammation. In patients with psoriasis, studies have reported a decrease in microbial diversity and a compositional shift characterized by an increase in *Staphylococcus aureus*, *Streptococcus sciuri*, and *Corynebacterium kroppenstedtii*, along with a decrease in commensals such as *Staphylococcus epidermidis* and *Cutibacterium acnes* [13,14]. These alterations appear more pronounced in lesional compared to non-lesional skin, suggesting a disease-related dysbiosis. *Staphylococcus aureus* promotes keratinocyte activation and IL-17-driven inflammation via superantigen and protease signalling. *Staphylococcus epidermidis* normally maintain barrier integrity by stimulating production of tight junction proteins (e.g., occludin, claudin-1) and antimicrobial peptides [15]. Their depletion in psoriatic skin compromises these protective functions. *Cutibacterium acnes* can produce metabolites that activate the aryl hydrocarbon receptor (AhR), promoting regulatory T cell differentiation and suppressing Th17-mediated inflammation [16]. This loss of beneficial commensals coupled with pathobiont expansion creates an immunologically hostile environment that perpetuates psoriatic inflammation. Additionally, multi-omics studies have identified microbial and metabolic biomarkers in psoriatic skin that correlate with disease severity and therapeutic response [16,17]. Moreover, interventional strategies such as the use of prebiotic creams, postbiotic molecules (e.g., bacterial lysates), and skin microbiota transplantation are under investigation. Emerging evidence suggests that restoring cutaneous microbial balance may synergize with systemic treatments, improving efficacy and possibly reducing relapse rates [18].

### 3.3. Oral Microbiota in Psoriasis

Although often overlooked, the oral microbiota may be altered in patients with psoriasis and psoriatic arthritis, and represent a potential inflammatory reservoir in the “oral-gut-skin” skin axis. Qiu et al. reported increased oral colonization by *Candida* species, especially in patients with more severe cutaneous involvement [19]. Recent quantitative analyses using 16S rRNA sequencing have revealed more detailed patterns: Zhao et al. (2024) demonstrated increased relative abundances of *Prevotella*, *Prevotella_7*, and *Porphyromonas gingivalis*, coupled with decreased *Haemophilus* in psoriasis patients compared to healthy controls [20]. They also found a positive correlation with *Alloprevotella*, *Porphyromonas*, and *Neisseria* with the severity of psoriasis (PASI score), while *Veillonella* showed a negative correlation [20]. At the phylum level, this manifests as increased *Bacteroidetes* and decreased *Proteobacteria*. Mechanistically, this oral dysbiosis could contribute to systemic inflammation via two pathways. First, the “swallowed microbiota” hypothesis suggests that oral pathobionts (e.g., *Porphyromonas*, *Klebsiella*) can translocate to the gut exacerbating intestinal dysbiosis and barrier permeability. Second, *Porphyromonas gingivalis* expresses peptidyl arginine deiminase (PAD), an enzyme capable of citrullinating host proteins. Citrullinated peptides are potent autoantigens that can trigger IL-23/Th17 activation, linking periodontal health directly to psoriatic inflammation [21]. Considering the frequent co-occurrence of periodontal disease and its potential role in amplifying Th17-mediated immune activation, the oral microbiota deserves greater attention as a potential modifier of disease progression and treatment responsiveness [22]. Particular attention should be pay when prescribing therapies that can increase the risk of candidiasis (e.g., IL-17 inhibitors).

**Table 1:** Summary of major taxonomic alterations and their functional immunologic consequences across gut, skin, and oral niches in psoriasis.

Site	Key Alteration	Effects	References
**Gut**	↓ *Bacteroidetes* (incl. *Bacteroides* spp.) ↓ *Akkermansia muciniphila* (mucin-degrading) ↓ *Faecalibacterium prausnitzii* (butyrate-producer) ↑ *Firmicutes* & Actinobacteria ↑ *Escherichia-Shigella* (pathobionts)	Impaired Barrier & Inflammation: Reduced production of SCFAs (acetate, butyrate) impairs barrier integrity and fails to induce regulatory T cells (Tregs). Expansion of pathobionts increases circulating microbial products (e.g., LPS), driving a Th1/Th17-dominant inflammatory profile and systemic inflammation (“leaky gut”)	[6,7,10,11]
**Skin**	↓ Microbial Diversity ↑ *Staphylococcus aureus* ↑ *Streptococcus sciuri* ↑ *Corynebacterium kroppenstedtii* ↓ *Staphylococcus epidermidis* ↓ *Cutibacterium acnes*	*S. aureus* promotes IL-17-driven inflammation via superantigen and protease signaling. Depletion of *S. epidermidis* compromises tight junction proteins (e.g., occludin) and AMP production. Loss of *C. acnes* reduces AhR activation, impairing Treg differentiation.	[12,13,14,15,16,17]
**Oral**	↑ *Candida* species ↑ *Prevotella*/*Prevotella_7* ↑ *Porphyromonas gingivalis* ↑ *Alloprevotella* & *Neisseria* (correlated with PASI)	“Swallowed microbiota”: Translocation of pathobionts (e.g., *Porphyromonas*, *Klebsiella*) exacerbates gut barrier permeability. *P. gingivalis* expresses PAD enzyme, causing protein citrullination; these autoantigens trigger IL-23/Th17 activation.	[21,22,23,24]

**Abbreviations:** SCFAs, short-chain fatty acids; Tregs, regulatory T cells; LPS, lipopolysaccharide; Th1/Th17, T helper type 1/17 cells; AMP, antimicrobial peptides; AhR, aryl hydrocarbon receptor; PASI, Psoriasis Area and Severity Index; PAD, peptidylarginine deiminase. **Legend:** ↑ increased; ↓ decreased.

## 4. Pharmacomicrobiomics in Psoriasis

### 4.1. Acitretin

Acitretin is a second-generation monoaromatic retinoid that exerts its therapeutic effect by binding to nuclear retinoic acid receptors (RARs) and retinoid X receptors (RXRs), thereby normalizing keratinocyte differentiation and reducing epidermal hyperproliferation [23]. Direct microbiome studies on acitretin monotherapy are still missing, representing a gap in our understanding of retinoid therapy in psoriasis. In a prospective study of 70 psoriasis patients, Zhou et al. treated one arm with acitretin plus a traditional “cooling-blood and detoxifying” formula (CBDF) and another with acitretin alone. After 8 weeks, the acitretin + CBDF group showed a significant increase in gut microbiota α-diversity compared with baseline, whereas the acitretin-only arm did not exhibit that gain in diversity [24]. This suggests that acitretin, when combined with adjunctive herbal therapy, can modulate the gut ecosystem, although the effect of acitretin monotherapy on microbial diversity was negligible [24].

### 4.2. Methotrexate

Methotrexate (MTX) is a folate antagonist that inhibits dihydrofolate reductase (DHFR), suppressing DNA synthesis in rapidly dividing cells and increasing the release of adenosine, a potent endogenous anti-inflammatory mediator [25]. Gut microbiota can markedly influence methotrexate (MTX) PK and, or PD. In vitro, human gut bacteria can metabolize/clear MTX, potentially reducing its therapeutic effect [4]. Studies on patients affected by rheumatoid arthritis (RA) found that baseline gut composition predicts MTX response [26]. Qiu et al. analyzed blood metabolomes and gut metagenomes in psoriasis patients before MTX. They found that baseline metabolic/microbial features predicted outcome: poor responders had higher levels of serum nutrient metabolites and a more “enriched” gut microbiota (e.g., *Bacilli*, *Lactobacillales*, *Burkholderiales*, *Gemella* spp. and *Bacteroides faecis*) [19]. In contrast, responders showed lower baseline microbial activity. After 16 weeks of MTX, the gut microbiome showed reduced metabolic pathway activity, and good responders exhibited higher remaining microbial activity but less fatty acid biosynthesis [19]. The authors conclude that the blood metabolic state and gut microbiota composition influence MTX effectiveness, and that MTX might exert part of its effects through the modulation of symbiotic intestinal bacteria [19]. Similarly, a review notes that psoriasis patients failing MTX tend to have greater gut microbial diversity and lower microbial metabolism compared to responders [27]. These observations imply that a highly diverse or active gut flora may reduce MTX efficacy, perhaps by metabolizing or otherwise neutralizing the drug. Although detailed pharmacokinetic studies are lacking in psoriasis, it is known from RA that gut bacteria can modulate MTX metabolism (e.g., bacterial dihydrofolate reductase variants) [27]. Thus, gut microbial profiling might help anticipate MTX response. Conversely, MTX treatment itself alters the gut microbiome [19]. The reduction in microbial metabolic pathways seen post-MTX suggests that MTX directly or indirectly suppresses certain bacteria (possibly those important for folate metabolism) [19]. In vitro, MTX can inhibit bacterial growth by blocking dihydrofolate reductase, and animal studies have shown that methotrexate reduces levels of certain gut commensals [27]. These effects may contribute to both efficacy and adverse effects (e.g., MTX-induced dysbiosis could relate to gastrointestinal toxicity). In summary, current evidence in psoriasis suggests a two-way interaction: gut microbiota composition influences MTX efficacy, and MTX perturbs the gut microbial ecosystem [19,27]. Identifying specific taxa or genes involved (e.g., folate pathway bacteria) remains an area for further study.

### 4.3. Cyclosporine

Cyclosporine A (CyA) is a calcineurin inhibitor that specifically targets T-lymphocytes. By forming a complex with cyclophilin, it blocks the phosphatase activity of calcineurin, preventing the translocation of NF-AT and subsequent transcription of cytokines like IL-2 [28]. CyA is a lipophilic drug with variable oral absorption. Preclinical data highlight a remarkable impact of gut microbes on CyA pharmacokinetics. In antibiotic-treated rats (microbiota-depleted), CyA bioavailability increased by ~155% [29]. This was accompanied by a significant downregulation of hepatic and intestinal CYP3A1, UGT1A1 and P-glycoprotein expression in the antibiotic group [29]. FMT restored microbial communities and normalized CyA levels. Correlation analysis identified specific genera (e.g., *Alloprevotella*, *Oscillospiraceae* UCG-005, *Parasutterella*, *Eubacterium xylanophilum*) whose abundance was linked to CyA bioavailability [29]. These results indicate that gut bacteria modulate CyA metabolism and transport: depletion of bacteria reduces drug metabolism (less CYP3A1) and efflux (less P-gp), raising blood levels of CyA. Though a preclinical study, it strongly suggests that interpatient variability in CyA levels, which is a clinical issue, might be partly due to microbiome differences. On the other side, studies in humans suggest that CyA itself has a limited effect on gut microbial composition. In a pilot ex vivo and in vivo study, healthy subjects received colon-targeted microencapsulated CyA (SmPill). The microbial diversity and relative abundance changed very little after 6 weeks of treatment [30]. Short-chain fatty acid analysis showed some increase in butyrate, but the overall community structure remained stable [30]. Therefore, unlike broad-spectrum antibiotics, CyA seems to exert minimal perturbation of the gut microbiota when delivered in a targeted formulation. In summary, preclinical data strongly support that the intestinal microbiota influences CyA pharmacokinetics, whereas clinical data indicate CyA (in microincapsulated formulation) does not markedly disrupt gut bacterial communities [29,30]. This suggests that baseline microbiome profiles could potentially predict CyA blood levels and risk of toxicity.

### 4.4. Biologics (Anti-TNF, Anti-IL-17, Anti-IL-12/23, Anti IL-23)

Biologic therapies target specific cytokines driving the psoriatic inflammatory cascade. TNF-α inhibitors (adalimumab, infliximab, etanercept, and certolizumab pegol) block a broad upstream inflammatory mediator, while newer agents target the IL-23/IL-17 axis more selectively. Specifically, IL-17 inhibitors neutralize the effector cytokines IL-17A (secukinumab, ixekizumab) and IL-17F (bimekizumab), or block the IL-17 receptor A (brodalumab). Upstream, IL-23 inhibitors (guselkumab, risankizumab, tildrakizumab) and the IL-12/23 inhibitor (ustekinumab) prevent the expansion and survival of pathogenic Th17 cells [31]. Biologic drugs for psoriasis are among the most effective treatment for psoriasis [32] and exhibit differential effects on the microbiome.

Regarding the direct impact of treatment on microbial communities, patients treated with adalimumab for 3 months showed no significant changes in gut microbiome diversity or overall composition compared to baseline [33]. Gut microbiota of psoriasis patients differed from healthy controls, but adalimumab therapy did not further shift major taxa over the short term [33]. This suggests that TNF blockade has limited direct impact on gut flora composition. However, microbiome data for other anti-TNF agents remain limited. In contrast, IL-17 inhibitors have a more pronounced effect. A longitudinal study of psoriasis patients found that secukinumab caused profound gut microbiome alterations, including increases in *Proteobacteria* (e.g., *Enterobacteriaceae*) and decreases in *Firmicutes* and *Bacteroidetes* phyla [34]. In other words, anti-IL-17 therapy disrupted the dominant anaerobic gut bacteria. Taxonomically, IL-17 blockade significantly increased *Bacteroides stercoris* and *Parabacteroides merdae*, while significantly decreasing butyrate-producing genera *Blautia* and *Roseburia* [34]. These shifts may influence inflammation: *Blautia* and *Roseburia* are known to produce anti-inflammatory SCFAs and modulate Th17/Treg balance [27]. Moreover, Yeh et al. observed that a baseline gut microbiota characterized by the presence of *Citrobacter*, *Staphylococcus*, and *Hafnia/Obesumbacterium* taxa may serve as a potential biomarker predictive of secukinumab response [34]. On IL-12/23 blockade (ustekinumab), gut changes were milder: one study found only an increase in Coprococcus after 6 months, without significant global shifts. Beyond the gut, available evidence on the cutaneous microbiome during biologic therapy remains limited. On the skin, Loesche et al. observed that during ustekinumab treatment, microbial divergence increased across body sites without re-establishing a consistent skin microbiome, suggesting that low-abundance taxa (e.g., *Lactococcus,* *Neisseria*) appear only sporadically [34,35]. The specific implications of these patterns are not fully understood, but enrichment of commensals that produce short-chain fatty acids and other anti-inflammatory metabolites (e.g., *Firmicutes* members) may be beneficial [27].

Beyond these drug-induced changes, a crucial aspect of pharmacomicrobiomics is the role of the microbiome as a predictor of therapeutic response. Distinct baseline gut microbiota profiles have been associated with clinical outcomes, suggesting a potential predictive value for biologic selection. In the secukinumab study, responders and nonresponders had distinct pre-treatment microbiomes. Moreover, Yeh et al. observed that a baseline gut microbiota characterized by the presence of *Citrobacter*, *Staphylococcus*, and *Hafnia/Obesumbacterium* taxa may serve as a potential biomarker predictive of secukinumab response [34]. Similarly, functional metagenomic pathways differ between responder groups: responders to IL-23 blockade—which targets the IL-23 axis with potentially longer drug survival [36,37]—showed enrichment of taurine/hypotaurine metabolism, whereas IL-17 responder microbiomes were enriched in antibiotic and amino-acid biosynthesis pathways [38]. These taxonomic and metabolic signatures could potentially serve as biomarkers for selecting optimal biologic therapy.

**Figure 3:**
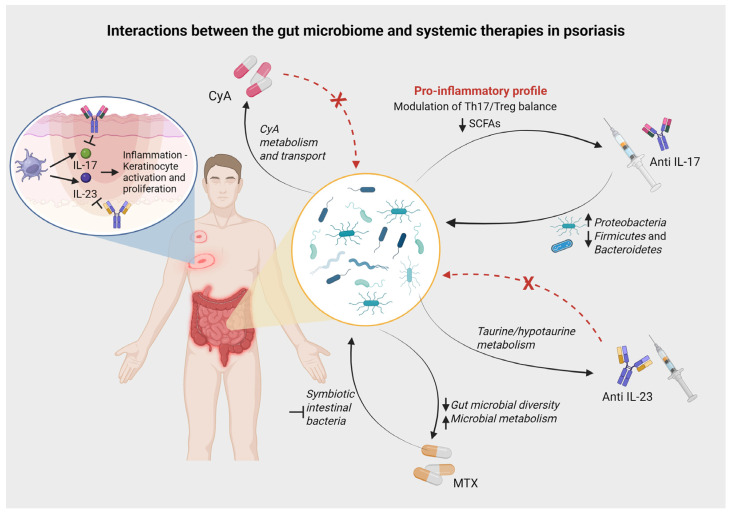
Schematic representation of the bidirectional crosstalk between the gut microbiome and systemic psoriasis therapies. The diagram highlights distinct pharmacomicrobiomic patterns across therapeutic classes. Different drugs exhibit variable interactions: Cyclosporine A (CyA) pharmacokinetics are modulated by gut microbial metabolism, yet the drug exerts minimal compositional pressure on the ecosystem. Conversely, Methotrexate (MTX) demonstrates a reciprocal relationship where bacterial enzymes degrade the drug, while treatment simultaneously reduces microbial diversity. Among biologics, Anti-IL-17 agents induce profound dysbiosis, characterized by an expansion of *Proteobacteria* and a depletion of beneficial *Firmicutes* and short-chain fatty acid (SCFA) producers, potentially fostering a pro-inflammatory environment. In contrast, Anti-IL-23 agents appear to maintain greater ecological stability, with therapeutic response linked to specific functional pathways (e.g., taurine metabolism) rather than broad taxonomic disruption.

The clinical implications of these bidirectional interactions are particularly evident in the context of safety and long-term maintenance of response. The depletion of beneficial commensals like *Blautia* and *Roseburia*, which modulate the Th17/Treg balance through anti-inflammatory SCFAs, may explain part of the therapeutic mechanism or, conversely, reduce drug efficacy in nonresponders [27,34]. Specifically, the reduction in butyrate-producing commensals observed during IL-17 inhibition may be mechanistically linked to the recognized risk of new-onset or exacerbated inflammatory bowel disease in these patients [11,39]. Overall, these data underscore a bidirectional interaction: biologics modulate gut bacteria, and the existing gut community modulates drug response (Figure 3). Importantly, while baseline gut microbiota signatures appear distinct between responders and non-responders, it remains to be determined whether these alterations are a direct driver of therapeutic resistance or a reflection of underlying disease severity and systemic inflammation. The clinical utility of microbiome-guided biologic selection remains an active area of research.

### 4.5. Small Molecules

Targeted small-molecule therapies in psoriasis (e.g., PDE4 and JAK inhibitors, fumarates) have emerging microbiome data. Apremilast, an oral phosphodiesterase 4 (PDE4) inhibitor that increases intracellular cyclic AMP levels, shows intriguing interactions with microbial flora. In a cohort of patients with recalcitrant psoriasis in difficult-to-treat areas such as nails, scalp, or intertriginous areas, apremilast therapy dramatically reduced fungal (*Candida*) colonization of skin, nails, and mucosa. After 52 weeks, 83% of patients with *Candida albicans* had complete clearance [40]. This suggests that by reducing inflammation (and Th17-mediated immune activation), apremilast may also restore microbial balance and prevent fungal overgrowth. Dimethyl fumarate (DMF) exerts its immunomodulatory effects primarily by activating the Nrf2 antioxidant pathway and suppressing NF-κB signaling, which reduces oxidative stress and inflammatory cytokine production. DMF is another systemic anti-psoriatic agent with microbiome effects, studied mainly in multiple sclerosis (MS). In MS patients, 6 months of DMF did not significantly change overall gut diversity, but did reduce abundance of certain *Clostridium* species [41]. Notably, patients experiencing gastrointestinal side effects and flushing had higher levels of *Streptococcus*, *Haemophilus*, *Lachnospira*, etc., and lower *Akkermansia*, implying that preexisting dysbiosis might predispose to intolerance [41]. These results imply that gut bacteria modulate DMF pharmacodynamics and side-effects. Janus kinase (JAK) inhibitors interfere with the JAK-STAT signaling pathway, blocking the intracellular transmission of signals from multiple cytokine receptors simultaneously (e.g., IFN-γ, IL-6) [42]. JAK inhibitors (tofacitinib, upadacitinib) have been less studied in psoriasis microbiomics. A small case series in psoriatic arthritis (PsA) treated with tofacitinib reported clinical improvement along with modest shifts in the gut microbiota. Overall bacterial diversity did not change significantly, but there was a relative increase in taxa considered immunomodulatory [43]. The authors concluded that tofacitinib selects for bacterial strains considered beneficial in immune modulation during treatment. Ex vivo analyses of human gut microbiota have provided mechanistic insights into the stability of these molecules. JAK inhibitors such as tofacitinib, baricitinib, and filgotinib appear resistant to microbial metabolism. Furthermore, they exert only minor effects on the overall composition and metabolic output of the human gut ecosystem [44]. Deucravacitinib is a first-in-class oral agent that selectively inhibits tyrosine kinase 2 (TYK2), a key mediator of cytokine signaling pathways downstream of the interleukin-23, interleukin-12, and type I interferon receptors [45]. To date, no studies have reported its interactions with the microbiome in psoriasis or other diseases [46]. In general, JAK inhibitors appear to have minor effects on gut flora [43], though human data are limited.

### 4.6. Probiotics

Probiotic and other microbiome-targeted interventions are being explored as adjunctive psoriasis therapies. In 2023, Buhas et al., in an open-label study, administered a spore-based Bacillus probiotic blend with fermentable oligosaccharides for 12 weeks alongside standard topical therapy; the supplemented group showed significantly greater reduction in PASI, DLQI and serum CRP levels, compared to controls. Gut microbiota analysis revealed shifts toward an anti-inflammatory profile: increased abundance of beneficial taxa (e.g., *Bifidobacterium*) and reduction of pro-inflammatory species [47]. These changes paralleled improved skin outcomes, supporting a therapeutic gut–skin axis effect. A meta-analysis of seven clinical trials found that probiotic supplementation significantly improves psoriasis outcomes. Although taking probiotics did not lower the risk of developing psoriasis, it significantly reduced PASI scores and increased the proportion of patients achieving PASI75 responses [48]. These findings suggest that reshaping the gut microbiome (e.g., increasing *Lactobacillus* and *Bifidobacterium*) can enhance anti-inflammatory pathways in psoriasis patients. Probiotics likely work by increasing beneficial microbial taxa that produce anti-inflammatory metabolites (such as SCFAs) and by strengthening the gut barrier, thereby reducing systemic inflammation. FMT has been trialed in few cases, with reported benefit [49]. Overall, the evidence supports microbiome adjunct therapy: targeting the microbiota can complement pharmacotherapy [50]. Importantly, probiotics appear safe: reported adverse events were mild (gastro-intestinal symptoms) and no serious events were attributed to probiotics [51]. Personalized use of probiotics or prebiotics to optimize drug response is an active research area. A summary of microbiome–drug interactions in systemic therapies for psoriasis is shown in Table 2.

## 5. Conclusions

The interplay between the microbiome and psoriasis systemic therapies is complex and may have significant clinical relevance. Emerging evidence indicates that gut and skin microbial communities can modulate drug efficacy and toxicity, while treatments, in turn, reshape these communities. For instance, baseline gut microbiota diversity and metabolic activity have been linked to methotrexate responsiveness [19], and pre-treatment gut signatures have predicted response to IL-17 blockade [27]. Likewise, systemic therapies induce characteristic microbiome shifts (e.g., IL-17 inhibitors reduce beneficial SCFA-producers) that could theoretically exacerbate inflammation or contribute to a pro-inflammatory environment [6].

From a practical standpoint, these findings suggest that microbiome profiling could evolve from a research tool to a clinically relevant complement to phenotype-based decision-making, potentially helping to refine therapy selection and sequencing in patients with difficult-to-treat disease or comorbidity risk. In parallel, adjunctive microbiome interventions (probiotics, FMT) have shown the capacity to improve psoriasis outcomes, likely by reinforcing anti-inflammatory microbial metabolites and barrier integrity; however, evidence remains limited and heterogeneous, underscoring the need for high-quality trials before routine adoption [47,51]. Importantly, it must be acknowledged that the current literature exhibits a significant imbalance, with the great majority of data focusing on the gut microbiota. This dominance likely reflects a research bias toward intestinal sampling and analytical maturity rather than biological primacy, as the oral and skin microbiota may also play critical, yet understudied, roles in the systemic inflammatory axis. Looking ahead, the most actionable biomarker direction is not a single taxon but reproducible multi-feature “signatures” integrating diversity metrics, functional pathways, and metabolite-linked immune readouts that can be measured longitudinally and mapped to drug PK/PD and clinical endpoints. To translate this paradigm into clinical practice, future research must prioritize longitudinal, multicenter studies with standardized microbiota collection and sequencing methodologies, and should integrate multi-omics data (metagenomics, metabolomics) with PK and clinical outcome measures. A critical goal is to identify actionable microbial biomarkers that reliably predict treatment response or adverse reactions across systemic therapies, supported by mechanistic validation of microbe–drug–host immune interactions and by randomized trials testing microbiome-guided adjuncts (for example, targeted probiotics administered alongside systemic therapy) to determine whether intentional microbial modulation can augment therapeutic efficacy or mitigate toxicity. In conclusion, pharmacomicrobiomics offers a novel precision-medicine perspective: integrating microbiome data with genetics and clinical factors may optimize treatment choice and predict therapeutic response. While not yet ready for routine clinical implementation, the concept that microbial metabolism, immune modulation, and barrier integrity influence drug efficacy and safety deserves rigorous exploration. In the future, baseline microbiome profiling may complement clinical and genetic data to guide the choice, sequencing, and combination of systemic therapies in psoriasis.

## Figures and Tables

**Table 2:** Summary of Microbiome-Drug Interactions in Psoriasis Systemic Therapies.

Treatment	Drug Class	Effects on Gut Microbiota	Effects on Skin/Oral Microbiota	Microbiota Effects on Drug PK/PD	Predictive Biomarkers	References
**Acitretin**	Retinoid	Minimal effect on diversity as monotherapy; increased α-diversity when combined with CBDF (8 weeks)	Not reported	Not reported	None identified	[26]
**Methotrexate**	Folate-antagonist	Reduces metabolic pathway activity; suppresses folate-metabolizing bacteria; dysbiosis-related GI toxicity	Not reported	Bacterial dihydrofolate reductase metabolizes/clears MTX, reducing efficacy; highly diverse/active flora decreases drug levels	Poor responders: ↑ baseline diversity, ↑ *Bacilli*, *Lactobacillales*, *Burkholderiales*, *Gemella* spp., *Bacteroides faecis*, elevated serum nutrient metabolites	[4,21,28,29]
**Cyclosporine A**	Calcineurin inhibitor	Minimal perturbation with colon-targeted formulation; stable diversity and SCFA levels (6 weeks); slight ↑ butyrate	Not reported	Gut bacteria modulate bioavailability via CYP3A1, UGT1A1, P-gp regulation; microbiota depletion ↑ bioavailability ~155%	*Alloprevotella*, *Oscillospiraceae* UCG-005, *Parasutterella*, *Eubacterium xylanophilum* abundance correlates with CyA levels	[31,32]
**Adalimumab**	TNF-α inhibitor	No significant changes in diversity or composition (3 months)	Not reported	Not reported	None identified	[35]
**Secukinumab**	IL-17 inhibitor	Profound alterations: ↑ *Proteobacteria* (*Enterobacteriaceae*), ↑ *B. stercoris*, ↑ *P. merdae*; ↓ *Firmicutes*, ↓ *Bacteroidetes*, ↓ *Blautia*, ↓ *Roseburia* (butyrate-producers)	Increased microbial divergence across body sites; transient low-abundance colonization	Baseline composition influences response	Baseline *Citrobacter*, *Staphylococcus*, *Hafnia/Obesumbacterium* predict response; distinct pre-treatment microbiomes in responders vs. non-responders	[29,36]
**Ustekinumab**	IL-12/23 inhibitor	Mild changes: ↑ Coprococcus (6 months); no significant global shifts	Greater baseline heterogeneity in lesional vs. non-lesional skin; increased divergence during treatment; sporadic low-abundance taxa (*Lactococcus*, *Neisseria*, *Acinetobacter*)	Not reported	None identified	[36,37]
**Guselkumab**	IL-23 inhibitor	Functional shifts: enriched taurine/hypotaurine metabolism in responders	Not reported	Baseline functional pathways influence response	Responders: ↑ taurine/hypotaurine metabolism (distinct from IL-17 responders with antibiotic/amino-acid biosynthesis enrichment)	[40]
**Apremilast**	PDE4 inhibitor	Not reported	Dramatic ↓ fungal colonization (skin, nails, mucosa); 83% *C. albicans* clearance (52 weeks)	Not reported	None identified	[42]
**Dimethyl Fumarate**	Fumaric acid ester (Nrf2 activator)	No significant diversity change; ↓ certain *Clostridium* species (6 months)	Not reported	Pre-existing dysbiosis predisposes to side effects	GI side effects/flushing: ↑ *Streptococcus*, *Haemophilus*, *Lachnospira*; ↓ *Akkermansia*	[43]
**Tofacitinib**	JAK1/3 inhibitor	Minor effects; stable diversity; modest ↑ immunomodulatory taxa	Not reported	Not reported	None identified	[45]
**Probiotics**	Microbial intervention	↑ *Bifidobacterium*, ↑ *Lactobacillus*; ↓ pro-inflammatory species; improved barrier integrity; enhanced SCFA production	Not specifically reported	N/A (direct modulation)	None required	[47,48]
**Fecal Microbiota Transplant**	Microbial intervention	Community restoration; case reports show clinical benefit	Not reported	N/A (direct modulation)	None established	[50,51]

**Abbreviations:** CBDF, cooling-blood and detoxifying formula; CYP, cytochrome P450; GI, gastrointestinal; IL, interleukin; JAK, Janus kinase; MTX, methotrexate; N/A, not applicable; Nrf2, nuclear factor erythroid 2-related factor 2; PD, pharmacodynamics; P-gp, P-glycoprotein; PK, pharmacokinetics; SCFA, short-chain fatty acid; TNF, tumor necrosis factor; UGT, UDP-glucuronosyltransferase. **Legend:** ↑ increased; ↓ decreased.

## Data Availability

No new data were created or analyzed in this study. Data sharing is not applicable to this article.
